# Social Dynamics Established Through Sport: Implications for the Sports Training of Young Brazilian Basketball Athletes

**DOI:** 10.3390/sports13030084

**Published:** 2025-03-10

**Authors:** Larissa Fernanda Porto Maciel, Sergio José Ibáñez, Mariana Klauck Beirith, Alexandra Folle

**Affiliations:** 1 Center for Health and Sports Sciences, Santa Catarina State University, Florianopolis 88080-350, Brazil; marianaklauck@outlook.com (M.K.B.); alexandra.folle@udesc.br (A.F.); 2 Training and Sports Performance Optimization Group, University of Extremadura, 10003 Cáceres, Spain; sibanez@unex.es

**Keywords:** interpersonal relationships, social support, youth sports, mixed-method research, pragmatism

## Abstract

This article aims to identify the influence of the social dynamics experienced by basketball players, specifically the type of support and help they received during their formative years in the sport. To this end, we conducted a mixed-method study with a sequential explanatory design. Quantitative data were collected from Brazilian athletes aged 18 and 19 (*n* = 141), followed by in-depth interviews with 24 athletes. The Kruskal–Wallis test was used to determine the differences between the age groups, and the association between the qualitative variables was analyzed using the Chi^2^ test, which was aided by the SPSS statistical program. Thematic analysis guided the coding and analysis of the qualitative data. The results showed that the family provided support at all formative stages, with the type and amount of the support supplied changing over time. Three types of support provided by family members were identified, with emotional and tangible support being more prevalent up to age 17. In contrast, informational support was more prominent in the final phase of sports training. Peers mainly provided emotional and informational support, especially from the age of 15. The results showed that the club, school, and coaches supported the athletes to keep playing basketball, usually through transportation, scholarships, food, and athlete grants. These findings provide valuable information on the dynamic nature of social relationships in the athlete development process. They also show that the support offered influences the wider environment to optimize or hinder athletic development in basketball. There is still a long way to go in understanding the social dynamics within the complex development processes in sports. Longitudinal studies with ecological, conceptual, and methodological approaches that provide a more integrative, complementary, holistic, and contextualized view of the influence of social dynamics on athlete training and sports participation could be an avenue to explore in future research.

## 1. Introduction

You only have to go through the autobiography of any notable athlete to discover that one of the main themes is social support. There will certainly be discussions about training tactics, victories and defeats, resilience, and glory. Still, the backdrop to all this will probably be the athlete’s support from important people throughout their career [1]. In the broadest sense, social support refers to “[…] social interactions designed to induce positive outcomes” [2] (p. 85). More contemporary conceptualizations have referred to social support as a multi-construct that encompasses structural (the number and type of relationships) and functional (interpersonal relationships) components [1,3].

Functional components refer to the specific purposes of structural relationships, a form of integration related to the number and types of ties and relationships in the athlete’s social network. The functional components can be further categorized into four specific forms of socially favorable behavior: emotional, esteem, informational, and tangible [4,5].

Emotional support refers to actions that convey a sense of being loved and cared for by others (e.g., encouragement, motivation, affection) [1,5]. Esteem support refers to actions that reinforce a sense of competence and ability (e.g., positive feedback, encouragement) [1,4,5].

Informational support refers to guidance in various situations (e.g., advice, tips), while tangible support refers to receiving material forms of instrumental assistance (e.g., financial, logistical support) [1,5]. The functional components and their respective dimensions of support are often subdivided into two perspectives: perceived support and received support [1,6].

Perceived support relates to the subjective perception that support is available and can be obtained if needed. On the other hand, the support received refers to the support received, i.e., actions of help and assistance provided by people present in the athlete’s life [1,7]. Thus, social support can refer to supportive relationships, but it can also include helping actions coming from people close to you to induce positive results for someone else [8].

It is essential to conceptualize social support as a complex and multivariate construct based on its dimensions (emotional, esteem, informational, and tangible) and perspectives (perceived and received), especially in sports [3]. This is because the role of socially supportive and influential people cannot be underestimated, as it is essential for young people’s involvement in various performance contexts, not just in sports but also at school, at home, and in the future, at work [9].

Although there are divergent philosophical bases regarding the fundamental objectives of youth sports [10], there is agreement regarding the significant role that social agents play in facilitating positive development objectives in young people [11,12]. The way in which family members, coaches, and peers interact with athletes can have important implications for the results obtained from their participation in sports [13].

Although the social environment in which sports training occurs is complex, there is evidence that parents play an especially important role in childhood and pre-adolescence. At the same time, coaches and peers become more influential with increasing age [14]. The mechanisms through which social support influences results, especially in sports, are widely debated. However, research into perceived support during training stages is limited [6].

In the early years (diversification stage), parents’ main role is to provide their children with support and appropriate opportunities to optimize their enjoyment and involvement in sports [10]. During this early period, parents are not only the main socializing agents of children’s experiences with sports but also the main models of character, attitudes, and behavior for their children [3,15].

The specialization stage is difficult to delineate, as it is characterized by change. These changes include a decrease in the number of sports/activities, deliberate play being replaced by deliberate practice, and gradual changes in the roles of parents (from direct to indirect involvement), coaches (from helper to expert), and peers (from stimulation/participation to meeting emotional needs) [16,17].

From this stage onwards, young people spend a significant amount of their time in sporting environments, and as a consequence, the family takes a back seat while coaches become experts and role models. At this time, the coach has more access and influence over the athletes than other influential agents, such as parents, teachers, and friends, whose impact is consistently strong from early to late adolescence [18].

The coach takes on a vital role of social support and becomes capable of influencing the stress–exhaustion relationship, career satisfaction, and athletes’ sporting experiences [19,20]. However, it is important to understand that parents and coaches impact sports involvement differently, especially in young athletes, and that both are equally crucial [10].

Social interaction can be divided into positive social experiences (e.g., social support) and negative ones (e.g., insensitive behavior, overcharging, and neglect), affecting athletes’ involvement and performance differently. In this way, an athlete’s relationship with their coach is one of their career’s most critical interpersonal aspects, as it can facilitate or hinder their development as a sportsperson [21].

In contrast, the transition to the investment stage can be marked by uncertainty and anxiety. Parents of elite athletes have reported that negotiating this role change is stressful and challenging, as they are no longer at the center of their child’s athletic world [22]. Despite this, parents can and must adapt to provide unconditional social support and a haven for young people as they develop their independence and pursue their sporting and life goals [11].

While the sports literature has predominantly focused on how adult social agents can shape youth sports participation, there is growing recognition that peer interactions represent an important means of generating positive outcomes. As young people progress through their sporting careers, they tend to rely less on adults and more on friends as a source of information, making peers increasingly influential, especially in adolescence [13,23].

Adolescence marks a significant shift in loyalty and closeness from family to peers. Participation in a group fulfills the desire to feel connected to others, provides an environment for sharing personal experiences and receiving encouragement about oneself, and serves as a reference for testing new identity-related skills [24].

By feeling connected, athletes demonstrate greater intrinsic motivation, commitment, and competence, as well as problem-solving skills, companionship, and teamwork [25,26]. In contrast, highly competitive environments can trigger discordant peer relationships that are closely linked to negative outcomes such as reduced group cohesion and athletic performance [24].

Based on this evidence, it is clear that social agents play unique roles, just as their influences are also unique. For example, the way coaches perform the key functions of instruction, selection, and management (in a collaborative, positive, tolerant way) can influence the athlete’s motivation. In contrast, the role of parents revolves strongly around support and facilitation, and the way in which this support is provided (unconditionally, positively, collaboratively) is also fundamental [3,17].

The role of peers involves friendship, cooperation, and reinforcement of rules/values among the group, and the way in which this role is fulfilled (altruistic, tolerant) is central to determining the athlete’s motivation. It is, therefore, important to understand that as an athlete’s career progresses, these roles tend to change [16]. To plan successful interventions and foster a long life in sports, these changing roles and their integral links with motivational influences must be constantly assessed [17].

In short, a large body of research supports the fact that quality relationships with family, coaches, and peers are determinants of positive experiences and outcomes in sports [14,27,28]. Although studies on the subject have attracted increasing attention, most of them focus on athletes from Australia [29], the United States [30], Canada [27,31], Portugal [32], and Asia [33].

However, the cultural origin, national system, and development status of sports in different countries affect the training of athletes and access to long-term sporting careers. Brazil has long faced difficulties in terms of a national system as a consolidated structure, which, combined with the scarce national incentive policies that allocate most of the financial resources to high-performance sports, restrict the development of sports and affect the training of young athletes in the country.

In contrast, in the United States, sports are encouraged and introduced in schools and, later, in universities. This is such a successful training model that talented athletes receive scholarships to attend college and represent universities in highly competitive and prestigious national competitions. This is a clear example of the impossibility of a fair comparison between different countries when it comes to athlete training and demonstrates the need for research into the impact of social dynamics on athletes from different cultural backgrounds, especially in Brazil.

In light of this evidence, we are unaware of any studies that have used a mixed-method approach and pragmatic vision to study the sporting careers of Brazilian basketball athletes. Therefore, this study aimed to identify the influence of the social dynamics experienced by basketball athletes, specifically the type of support and help received by athletes over the years of their sports training.

## 2. Materials and Methods

### 2.1. Research Design

**Figure 1:**
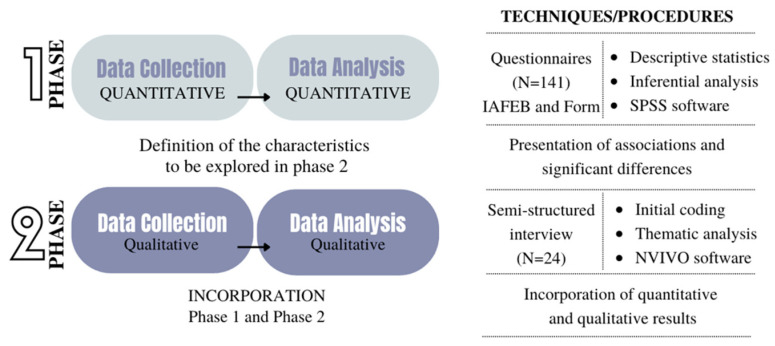
Diagram representing the sequential explanatory design. Note: IAFEB—Instrument for Evaluating Sports Training in Basketball.

This study used a two-phase sequential mixed-methods explanatory approach [34], conducted under pragmatic guidance [35]. The initial phase consisted of collecting and analyzing quantitative data, followed by a second phase that used qualitative methods to delve deeper into the themes with a selected group of participants (Figure 1). The study was approved by the Ethics Committee for Research Involving Human Beings of Santa Catarina State University (CEPSH/UDESC, opinion 4.733.011). All participants gave their informed consent before participating in the two research phases.

### 2.2. Quantitative Stage

#### 2.2.1. Participants and Eligibility Criteria

The first phase of the study was carried out with 141 basketball players aged 18 and 19 (78.7% male and 21% female), recruited through convenience sampling (non-probabilistic intentional) [36]. To be eligible to participate in the quantitative phase of this research, participants had to compete in Brazil in the year of data collection. This criterion was used to ensure that the participants involved in the interviews had sufficient experience to provide an in-depth perspective on the process of sports training and competition experience in Brazilian reality.

#### 2.2.2. Instruments

Two instruments were used in this study phase: the Instrument for Evaluating Sports Training in Basketball—IAFEB and the athletes’ characterization sheet. The IAFEB was adapted from the original version created by Collet et al. [37] and underwent two validation stages (content validity, pilot study) before it was applied.

**Figure 2:**
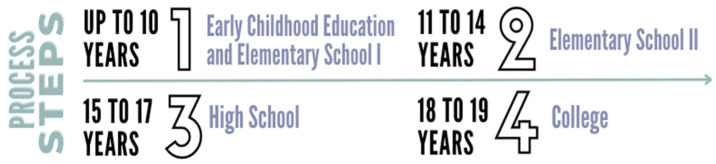
IAFEB sequential age groups, according to the compulsory stages of basic education and higher education in Brazil.

The development of the IAFEB was based on the three dynamic elements (personal engagement in activities, quality social dynamics, appropriate scenarios) outlined by the Personal Assets Framework—PAF [13,28,38] and is divided into sequential age groups, according to the compulsory stages of basic education and higher education in Brazil:

(I) Eight items refer to the activities practiced in school physical education;
(II) Forty-six items are related to sports activities, subdivided into four parts, as illustrated in Figure 2.

The characterization form was designed specifically for this study to collect the athletes’ personal information (eight items) and sports information (nine items).

#### 2.2.3. Procedures

The questionnaires were assembled in electronic format using the Google Forms application. Firstly, the survey was sent by e-mail and via social media to the state federations and affiliated clubs, available on the website of the Brazilian Basketball Confederation (CBB—https://www.cbb.com.br/federacoes (accessed on 15 April 2023)). Social media were created (Instagram, Twitter, WhatsApp) to share the link to access the electronic tool and publish the folder promoting the survey. Thirdly, the academic community, coaches, athletes, and sports fans were asked to share the research to increase the study’s visibility nationwide. Finally, the fourth strategy involved snowball sampling, selecting participants based on referrals from previous interviewees [39].

The athletes participated in this research phase after being informed of the study’s objectives and procedures and after reading and signing the (TCLE). On agreeing to participate, the athletes were asked to fill in the e-mail address to which a copy of the agreement had been sent and then to fill in the electronic instrument available on Google Forms. When the questionnaire was completed, the information was automatically forwarded to a spreadsheet generated by the application and sent to the e-mail address created specifically for the study. The time taken to complete the questionnaire was between 15 and 20 min. The information was collected from June to December 2021.

#### 2.2.4. Statistical Analysis

All data were recorded and analyzed using SPSS 24.0 statistical software (SPSS Inc., Chicago, IL, USA), with a statistical reference value of *p* < 0.05. Quantitative variables were summarized with descriptive statistics using mean and standard deviation, while qualitative analyses were compared with relative and absolute frequency. Criteria assumption tests (normality test) were carried out using the Kolmogorov–Smirnov test. The Kruskal–Wallis test was used to determine the differences between the age groups, and the association between the qualitative variables was analyzed using the Chi^2^ test.

### 2.3. Qualitative Stage

#### 2.3.1. Participants and Eligibility Criteria

The second phase of this study involved 24 athletes (70.8% male and 20.1% female) recruited after the quantitative study’s results [39]. Following the assumptions described by Creswell and Plano Clark [40] for choosing participants in the qualitative phase in a mixed-method study of the sequential explanatory type, we sought to select at least two athletes from each region of the country.

The following criteria were used to select the athletes in the qualitative phase:

(I) Athletes called up to the national team;
(II) Athletes called up to the state team;
(III) Athletes with more time spent practicing basketball.

**Figure 3:**
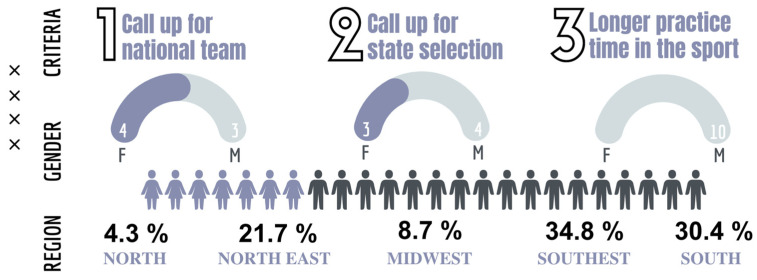
Diagram representing the inclusion of athletes in the qualitative stage.

In the Brazilian regions where no athletes of both sexes participated, two athletes of the same sex were selected. The first athlete was selected based on criteria I and II, while the second athlete was selected according to criterion III, in order to obtain different evidence about the reality of the sports training of athletes within the same region/state (Figure 3).

When an athlete who could not be found was selected, another athlete was immediately selected, and, if necessary, other athletes were invited to participate in the second phase of the research, regardless of the criteria. To include athletes from all over Brazil, in regions where only one athlete took part in the quantitative stage, they were invited to participate in the qualitative stage, regardless of the selection criteria.

#### 2.3.2. Instruments

The individual interviews were conducted with questions about the athletes’ relationships with the people closest to them, such as their incentives to continue in the sport, their relationships with family, coaches, and friends, and the financial support they received to train and compete. The questions were formulated following the dynamic element outlined by the PAF (quality social dynamics) [13,28,38]. A pilot study was carried out to verify the specificity of the interview script and the quality of the results generated [41].

#### 2.3.3. Procedures

Each athlete was interviewed separately. Based on the information obtained in the first phase of the study and the snowball strategy, the athletes selected for this stage of the research were contacted via e-mail, social media, and WhatsApp messages. A maximum of four contact attempts were made with each selected athlete at different times.

The athletes took part in this phase of the research after being informed of the objectives of the study, ensuring the confidentiality and anonymity of their participation, and after reading the Free and Informed Consent Form—TCLE for this phase of the study and the Consent for Photographs, Videos, and Recordings—CFVG. After accepting the TCLE and the CFVG, each athlete individually scheduled a day and time for the telephone interview, which took place between April and July 2022.

The interviews were audio-recorded and later transcribed. Once transcribed, the main author read the interviews while listening to the recordings to ensure the accuracy of the transcriptions. The material was sent to the athletes so that they could validate the transcribed content, and only two requested changes to the transcribed material, which were promptly complied with. The average duration of each interview was one hour. NVivo qualitative data analysis software for Mac (version 10.2.2 ©QSR International) was used to organize and analyze the data. The data were de-identified to ensure confidentiality, and each participant was given a number.

#### 2.3.4. Qualitative Analysis

Data analysis was guided by the thematic analysis technique with a deductive design [42]. The qualitative data were analyzed using the six stages proposed by Braum and Clark [43], which are summarized as follows: (I) familiarization with the data by reading the transcripts; (II) generation of initial codes using a deductive approach (guided by theory); (III) construction of themes with the help of visual mapping and continuous involvement with the data; (IV and V) reviewing and defining the themes and exporting the initial codes and their associated excerpts to specific software. Finally, the final report was produced (VI).

#### 2.3.5. Reliability

To guarantee the quality of the analysis and the reliability of the data, the critical friend strategy was adopted, with the aim of encouraging introspection on the part of the main researcher through reflective dialogs with the members of the research team [44]. Therefore, the analysis process involved the participation of two researchers, enabling alternative interpretations of the data, in addition to feedback and self-reflection from the main researcher, which led to small refinements of the thematic scheme.

The results were pooled to produce a more complete picture and avoid the biases inherent in using a monomethod design [45,46], using the results from the analysis of one form of data (quantitative) to address the analysis of another form of data (qualitative). That is, the athletes’ narratives are provided in the Results Section to support the interpretations of the quantitative data.

## 3. Results

### 3.1. Type of Support Received from Those Closest to Them

The results shown in Table 1 demonstrate the influence that family has on all ages, providing greater emotional support from 15 to 17 years old (38%), informational support from 18 to 19 years old (60%), and tangible support from 15 to 19 years old (33%). Coaches and peers begin to have a greater influence from age 15, but the significant results show greater informational and emotional support from peers (33%).

The narratives below illustrate the athletes’ perception of the social support they received, especially from their mothers, as a facilitator for their involvement in sports. The athletes’ comments also reflect the importance of teammates, both for the sporting context and for personal relationships beyond sport:

*“[…] thanks to my mother I was always able to train more, I had money to catch a bus, to eat something on the street, but there were girls who didn’t have that condition, they didn’t have a family, with a good financial condition to help them […]” (Athlete 8).*

*“[…] my mother was the person who encouraged me a lot. I was going through a difficult time in my life; my parents had split up, my grandparents had died, I was having problems at school, and some behavioral problems, and basketball started to help me a lot. When I didn’t want to play anymore, that was when my mother encouraged me the most. She supported me even though I wasn’t in the right frame of mind. Those were the most important moments that perhaps made me dedicate myself even more […]. I felt better, which was very important; the support she gave me from an early age was very important” (Athlete 59).*

*“It’s always been my parents who have supported me in every decision I’ve made, in every decision I’ve taken, it’s always been them who have supported me, who have talked to me” (Athlete 7).*

*“My family paid for everything. Fortunately, I lived next to the court, so I didn’t have to spend any money” (Athlete 5).*

*“My family helped me a lot financially, with materials to improve my performance and motivation. There were matches that were shown live on TV, and they recorded them. Their reaction to me playing in the league was nice to see. So I think moral and financial support were the main things my family helped me with […]” (Athlete 88).*

*“[…] I was wonderfully welcomed at the club; to this day, my personal friends are the ones who played with me when I was 14, so for me, it was wonderful […]” (Athlete 102).*

*“My friends, these friends who are also in [project name], have been my friends since I started playing. I started playing with them. It was a process of evolution with them” (Athlete 125).*

*“[…] the players helped me a lot with this, giving me tips and so on […]” (Athlete 33).*

### 3.2. Support Received During Sports Training

The results showed that, from age 15, most of the athletes began to receive some financial assistance to play basketball. Until age 14, few athletes received this type of support to maintain their involvement in the sport (Table 2).

When asked whether they had received any tangible support other than access to family resources during their sporting education, the athletes interviewed revealed that they had received no funding until they were around 14 years old. However, it can be seen that participation in sport competitions is among the criteria that enabled athletes to receive financial support for their sporting careers:

*“Not really. I used to cycle there, and I didn’t have any support, no grant, nothing; I just went for the passion of basketball” (Athlete 88).*

*“No, I never did. The only thing I received was the grant from the club itself; we had to pay the monthly fee, and I didn’t pay it” (Athlete 7).*

*“From U16 onwards, when I managed to make the Brazilian team and play in the Copa America, I started to receive a salary from the Club” (Athlete 32).*

*“[…] until I was 14, it was on scholarship. From the age of 15 […] I did start to get paid. I didn’t sign a contract; it was more of an agreement with the coach, the president, and the team director to receive money to help me […] I started getting paid from the age of 14 to 15” (Athlete 59).*

*“[…] I really started to get help to train when I moved and went to play for Paraná when I was 17. From 13 to 17, it was my family who supported me financially […]” (Athlete 19).*

### 3.3. Type of Support Received During Sports Training

**Figure 4:**
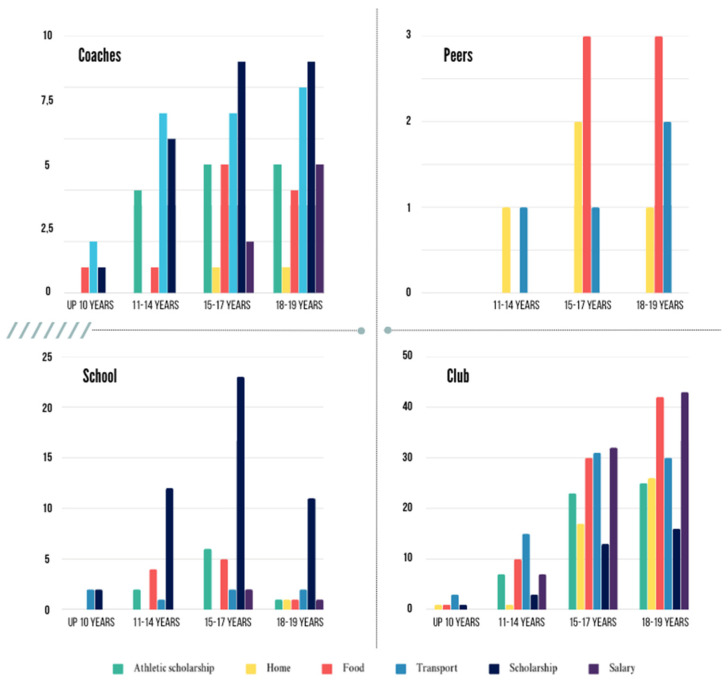
Type of support received during sports training.

Regarding assistance received during sports training, the club is the leader in offering transportation for players up to age 10. From age 15 onwards, other types of support, such as food, salary, and athlete grants, become more frequent (Figure 4).

The results showed that the club and, by extension, the coaches provided support to help athletes continue their sporting careers. The school offered a scholarship, while colleagues helped with food, housing, and transportation when necessary.

*“Yes, they [the club] paid for my tickets and accommodation, as well as my food and salary” (Athlete 98).*

*“At first, it was just for cost help. But then the 2021/2022 NBB season started, and I started getting paid minimum wage” (Athlete 2).*

*“They [the club] helped with transportation, with bus passes […]” (Athlete 90).*

*“I received housing, full meals, and a 400 reais allowance” (Athlete 33).*

*“I ended up getting a scholarship at a private school. The teams I played for always gave me a scholarship at a private school. So sport has paved the way, opening doors for me to improve my life” (Athlete 87).*

## 4. Discussion

This study aimed to expand the body of literature examining the influence of the social dynamics experienced by basketball athletes and to deepen our understanding of how the type of support and help received contributed to the athletes’ development over their years of training in the sport. The results showed that the family provided support at all formative stages, with the type and amount of the support supplied changing over time. Three types of support provided by family members were identified, with emotional and tangible support being more prevalent up to age 17. In contrast, informational support was more prominent in the final phase of sports training. Peers mainly provided emotional and informational support, especially from the age of 15. The results showed that the club, school, and coaches supported the athletes to keep practicing basketball, especially helping with transportation, scholarships, food, and athlete grants.

These findings offer relevant information about the dynamic nature of relationships between family, coaches, teammates, and athletes in youth sports and how these relationships operate in the wider sporting environment to optimize and sometimes hinder athletic development. As it is a conceptual model capable of explaining the mechanisms and results that constitute the positive development of young people, the Personal Assets Framework (PAF) was chosen for this study. Although there are various models, the PAF describes three dynamic elements that contribute to optimal long-term development in young athletes: personal engagement in activities, quality social dynamics, and appropriate contexts [28,38,47]. The second element was used to contextualize the findings of this study.

According to this framework, when sports training is designed appropriately for the development of athletes, the interaction of these three elements provides a positive experience that, when repeated over time, generates changes in the personal assets of young people. It is a structure that aims to understand young people’s experiences with sports over time through personal (what), social (who), and contextual (where variables [13,28].

The role of parents is composed of positive aspects, such as availability (physical presence and provision of support), interference (requested), and encouragement (motivation and demonstration that sports practice is valued by them), and negative aspects, such as interference (unrequested) and restrictions on practice [48]. For this reason, parental education programs in sports are increasingly necessary, because the more parents understand their role and their limits in the formative process of their children, the greater the chances of success in the short, medium, and long term [49]. Directive interventions are expected to promote more effective and positive parental involvement in sports to the point of benefiting athlete development and alleviating pressures on developing athletes [49,50].

Our findings reinforce other findings in the literature suggesting that parents influence an individual’s athletic development [14,27,51]. Consistent with the research carried out by Lima et al. [52] with handball athletes and Motta et al. [53] with squash athletes, we found the predominant existence of emotional and tangible support provided by parents during most athletes’ sporting careers. In a review carried out by Maciel et al. [14], evidence showed that support received from family was predominantly emotional. According to the authors, this type of support provided by family was pointed out as a fundamental factor in motivating athletes to practice sports, showing that, in general, they were present at training sessions and competitions and believed in the benefits of sports.

Support in the form of advice and tips provided by parents became more consistent as the athletes progressed to higher levels of competition and came closer to adulthood. Different results were found in research carried out with athletes from various sports, which documented greater informational support received by athletes in childhood and early adolescence [25] when parents usually take on roles as volunteer coaches to help their children in the early years of their involvement with sports [27]. Despite the relevance of this result, future research should look into this area in more detail to provide a more robust understanding of this specific type of support and its influence on the athlete’s development, especially during the transition to adult competitions.

Unlike informational support, tangible support from family proved consistent throughout the formative years. A different result was found by Lundy et al. [27], who emphasized that athletes received more tangible support in the early years of their involvement in sports. Similarly, a lower level of tangible support, especially after age 16, was reported by the basketball athletes investigated by Reis et al. [54]. Unlike other studies, our results suggest that even in the final stage of the formative process, parents continued to provide tangible support to their children. This means that although sports institutions traditionally make financial resources available to athletes at this stage, these are insufficient for them to devote themselves fully to basketball without financial help from their families.

In light of this evidence, despite the growth, there is still limited and generalized data on different populations of athletes since the models do not take into account the financial constraints of different components (public education system, social projects, sports clubs, social class, etc.) or that not all athletes have access to the same resources and practice possibilities [55]. Similarly, the existence of thousands of municipalities, each with its policy, the absence of a national database, and the lack of reliable data make a comprehensive survey of Brazilian sports difficult [56]. Therefore, government policies and economic support are factors that affect the development of athletes’ careers. Future research could identify the implications of tangible support and how this specific type of support has shaped the development of future sportspeople in the country.

The support provided by peers was predominantly emotional and informative, especially from age 15 onwards. This evidence corroborates the idea that as sports practice progresses, teammates’ roles are reshaped and become increasingly important, especially in the phases of specialization and sports investment [14]. According to Lima et al. [52], athletes undergo changes in their behavior, which, according to Mosher et al. [57], can occur due to influences close to or far from the athletes. Thus, social agents play unique roles, and their influences are also unique, i.e., as the athletic career progresses, these roles also tend to change [14,17].

In the review by Maciel et al. [14], the studies showed that the emotional and informational support received from colleagues contributed to the quality of athletes’ experiences in sports and were the two most prominent supports over time. Specifically, emotional support was more present, while the participants did not perceive tangible support. Consistent with these results, Lima et al. [52] reported that the social bonds established between athletes and their teammates contributed to their staying in sport, and, in the absence of parents, teammates were like a second family and offered all kinds of support. This is remarkable, considering the evidence that parents are the main providers and interpreters of youth sports, while teammates become important family representatives throughout sporting careers [58].

In addition to the financial resources received from family members, the results showed that the club, school, and coaches were the main providers of some kind of help for the athletes to keep playing basketball. The athletes reported receiving aid through transportation, scholarships, food, and athlete grants. Previous research carried out with basketball athletes [59] and athletes from different sports [60] has shown that one of the reasons leading athletes to specialize in sports, in addition to athletic success, is obtaining scholarships offered by their clubs. Traditionally, clubs establish partnerships with colleges and universities, most of which are private, and offer athletes the chance to continue their studies alongside their sporting development, usually to attract young talent.

The athlete grant usually takes the form of money, as a resource from the club and as an extension from coaches, municipalities, or even directly from the government. The source of funds is sometimes confusing for athletes, but what really matters is being appreciated for all the time they put in and obtaining the financial stability that makes it possible to pursue the dream of a sporting career. In the absence of this support, athletes start looking for other alternatives to contribute to their family structure and, in the absence of these, end up abandoning sports altogether. In Brazil, the Athlete Grant Program does not follow a targeted investment policy. However, it is an important public policy for the Brazilian scenario, as it is the main form of individual investment for athletes [61].

This study does not aim to draw definitive conclusions on this subject but to problematize the extent to which this issue affects the sports training process of athletes in Brazil. Given this, we recognize the limitations of this research and reinforce the need for further studies that seek to identify the implications of the lack of scholarships (academic and sporting) for promoting sports in the country. No organized university sport provides athletes with adequate athletic and academic training. Joint work between academic, sports, public, and private institutions is necessary for athletes to undergo training (school and sports) and reach their full potential in their personal and professional lives.

Based on the above, the social dynamics experienced by athletes within the sporting context can range from dyadic relationships to team-based constructs and the wider organizational environment [46]. Recognizing the tautology and interaction between these varying levels is necessary when trying to understand the impact that social dynamics have on young people’s experiences of getting involved in sports [62]. Given the relative consensus regarding the potential benefits of practicing sports for children and adolescents while recognizing the problems related to the extensive web of dynamic and reciprocal relationships that define the youth sports system [58], as well as culture, regionality, access, and professionalization, this study sought to provide evidence on the elements that influenced the sports training of basketball athletes, in an attempt to understand the nature of the factors within a dynamic aspect of the PAF.

Although our findings offer important information and nuances regarding the type of support provided by social agents during the course of an athletic career in basketball, perhaps the most innovative contribution of the study is the understanding of the social dynamics obtained from the perception of athletes who were at the end of the training process in the sport, coupled with the effectiveness of a mixed-method approach that enabled greater involvement with the data and important elements shared by the athletes in the two phases of the research. Our findings also draw attention to the role of financial support in facilitating sports development, which can increase opportunities for young people to prolong their involvement in sports and thus compete in their desired sport at a competitive level.

## 5. Limitations and Future Directions

Before concluding, we should acknowledge some limitations of this study. Firstly, data collection occurred in only one country, and it is not possible to correlate data across cultures and settings. Secondly, although the athletes taking part in the quantitative phase were recruited from all over Brazil, we applied non-probabilistic sampling techniques, so it is not possible to rule out the occurrence of selection bias. Thirdly, due to the use of a retrospective questionnaire, we cannot completely exclude the occurrence of memory bias and social desirability bias. Furthermore, as the qualitative study consisted of a convenience sample recruited on the basis of pre-established criteria, our findings may not be generalizable to athletes from other countries, sports, and socioeconomic statuses. Finally, our number of participants is acceptable for qualitative interpretation but low for statistical analysis, i.e., a larger number of participants could have revealed new themes or provided additional context for existing themes in the same way that not conducting a longitudinal study prevents an assessment of the long-term effects of social support.

There is still a long way to go in understanding the social dynamics within the complex development processes in sports. Ongoing research into the influence of family, coaches, and peers on sports should go beyond the types of support provided and examine the relations between contextual factors (socioeconomic status, values) and the support perceived and received from these agents for young people’s participation in sports. It may be necessary to focus explicitly on each social agent to fully unravel the factors influencing social dynamics in sports and the additional relations that help or hinder athlete development. In addition, longitudinal studies with ecological conceptual and methodological approaches that provide a more integrative, complementary, holistic, and contextualized view of the influence of social dynamics on athlete training and sports participation could be an avenue to explore in future research.

## 6. Conclusions

Our results showed that the family provided support at all formative stages, with the type and amount of support supplied changing over time. Three types of support provided by family members were identified, with emotional and tangible support being more prevalent up to age 17. In contrast, informational support was more prominent in the final phase of sports training. Peers mainly provided emotional and informational support, especially from the age of 15. The results showed that the club, school, and coaches provided some kind of support for the athletes to keep practicing basketball, especially help with transportation, scholarships, food, and athlete grants.

This research presents findings that can be used to develop educational programs aimed at preparing parents and family members for their children’s sports development. These programs can ensure that each individual understands their role and adopts positive attitudes during the process, with the aim of better contributing to the development of young people, based on an understanding of the needs that athletes have at each stage of their development. Despite this, given the importance of coaches’ support in the training process, the study’s findings highlight the need for coach education programs that recognize their influence on the development of athletes. In addition to technical, tactical, and physical aspects, these programs should also emphasize motivational, psychological, and emotional factors, recognizing that a positive and meaningful sports experience can favor the continuation or discontinuation of a sports career in the long term.

## Figures and Tables

**Table 1:** Type of support received during sports training.

Training Stage	No Incentive	Informational	Emotional	Tangible	Chi^2^
	Parents				
Up to the age of 10	3 (6%)	6 (5%)	26 (16%)	15 (7%)	x^2^ = 70.04 *p*-value = <0.001
11 to 14 years old	11 (22%)	15 (14%)	51 (32%)	52 (26%)	
15 to 17 years old	18 (36%)	20 (19%)	62 (38%)	68 (33%)	
18 and 19 years old	18 (36%)	62 (60%)	20 (12%)	68 (33%)	
	Coaches				
Up to the age of 10	4 (18%)	30 (8%)	11 (15%)	5 (7%)	x^2^ = 12.51 *p*-value = 0.186
11 to 14 years old	6 (27%)	84 (24%)	25 (35%)	14 (19%)	
15 to 17 years old	6 (27%)	118 (34%)	18 (25%)	26 (37%)	
18 and 19 years old	6 (27%)	118 (34%)	18 (25%)	26 (37%)	
	Peers				
Up to the age of 10	13 (14%)	9 (6%)	25 (9%)	3 (3%)	x^2^ = 19.47 *p*-value = 0.021
11 to 14 years old	31 (34%)	29 (19%)	66 (25%)	3 (3%)	
15 to 17 years old	23 (26%)	56 (37%)	87 (33%)	2 (2%)	
18 and 19 years old	23 (26%)	56 (37%)	87 (33%)	2 (2%)	

x^2^—Chi-square.

**Table 2:** Financial support received during sports training.

Financial Support	Up to 10 Years	11 to 14 Years	15 to 17 Years	18 to 19 Years Old	Chi^2^
No	92%	79.8%	58.4%	51.8%	x^2^ = 50.28
Yes	8%	20.2%	41.6%	48.2%	*p*-value < 0.001

x^2^—Chi-square.

## Data Availability

The datasets employed in this study can be obtained from the corresponding author upon reasonable request. However, certain data cannot be made publicly accessible due to privacy considerations.
